# Public health system credibility and corporate financial risk: behavioral channels from China

**DOI:** 10.3389/fpubh.2026.1791324

**Published:** 2026-03-26

**Authors:** Caijie Zhou, Chia-Hsien Tang

**Affiliations:** College of Business, Guangxi Minzu Normal University, Chongzuo, Guangxi, China

**Keywords:** public health system credibility, corporate financial risk, stock price crash risk, behavioral responses, China

## Abstract

**Introduction:**

Public health systems influence economic behavior by shaping information credibility and risk perception during health emergencies. While existing studies primarily focus on infection severity and epidemiological outcomes, less attention has been given to the economic implications of public health system credibility.

**Methods:**

Using a sample of Chinese listed firms from 2017 to 2024, this study combines firm-level stock market data with regional indicators of public health system credibility. Firm-level financial risk is measured using stock price crash risk and downside volatility.

**Results:**

The empirical results show that declines in public health system credibility are associated with higher levels of firm financial risk.

**Discussion:**

Additional analyses indicate that employee behavioral withdrawal and reductions in consumer demand are related to this association. These findings suggest that credibility in public health communication may have implications for corporate financial stability beyond direct health outcomes.

## 1 Introduction

Public health systems play a central role in managing uncertainty during health emergencies. Their function extends beyond the provision of medical services. They also serve as key information providers that shape risk perception and guide behavior at both individual and organizational levels. A growing body of research shows that trust in health authorities is closely related to public compliance and social coordination during health emergencies (1, 2). When such trust is weakened, uncertainty intensifies and behavioral responses become more volatile.

Prior studies in public health emphasize that institutional trust is essential for effective crisis management. Transparent communication and consistent policy signals help stabilize expectations and reduce panic, thereby encouraging cooperative behavior (1, 3). In contrast, frequent data revisions, conflicting messages, or abrupt policy reversals may erode confidence in health systems (4). These credibility problems can alter how individuals interpret health risks and respond to official guidance, even when objective conditions remain unchanged.

Behavioral responses to health-related uncertainty have been widely documented. During epidemic outbreaks, individuals often reduce mobility, avoid workplaces, and postpone consumption, even in the absence of formal restrictions (5, 6). Importantly, these responses are not driven solely by infection rates or mortality risks. Public health research suggests that Public health research also suggests that the perceived credibility of health information influences individual behavior (7, 24). When information is unclear or contested, individuals rely more heavily on personal judgment, which may amplify withdrawal behavior.

Health shocks also generate substantial economic consequences. Existing evidence links epidemics and pandemics to lower economic growth, labor market disruptions, and productivity losses (8, 9). At the firm level, health-related disturbances affect production continuity, supply chains, and demand conditions (10). These effects often arise indirectly through changes in behavior rather than through direct medical costs or physical damage to firm assets.

Financial markets are particularly sensitive to uncertainty and information quality. A large finance literature documents that unexpected shocks increase volatility and downside risk (11, 12). During health crises, stock markets frequently experience sharp declines and heightened risk, especially in the early stages of outbreaks (13, 14, 26). Event-based studies further show that market reactions are influenced not only by reported infection numbers, but also by policy announcements and public signals issued by authorities (15).

Although prior research links pandemics to stock returns and volatility across countries (10, 16), most studies focus on the severity of outbreaks, such as confirmed cases or death rates. Comparatively little attention has been paid to the characteristics of public health systems themselves. Research on universal health coverage suggests that stronger health systems can mitigate economic vulnerability during health shocks (17, 18). However, the role of public health system credibility and trust as sources of firm-level financial risk remains underexplored.

Public health credibility refers to the perceived reliability, consistency, and transparency of information provided by health authorities. It differs conceptually from health severity and policy stringency. Health severity reflects the objective intensity of a health shock, while policy stringency captures the restrictiveness of government interventions. Credibility, by contrast, concerns the quality of information and governance signals rather than the magnitude of the health threat itself. Even under moderate health conditions, inconsistent reporting or frequent policy reversals may weaken confidence and increase uncertainty. Conversely, transparent and stable communication can anchor expectations during severe outbreaks. From an economic perspective, credibility therefore represents an institutional information shock rather than a proxy for disease intensity.

From an organizational perspective, credibility shocks affect firms primarily through labor and consumer channels. Perceived health risk influences employee absenteeism, job engagement, and productivity (19, 20). On the demand side, health concerns reduce in-person consumption and increase precautionary behavior among consumers (21). At the firm level, these behavioral responses are transmitted through internal organizational processes rather than through immediate market adjustment. Employee withdrawal disrupts production schedules, delays project execution, and weakens internal monitoring, making it more difficult for managers to assess short-term performance accurately. On the demand side, irregular sales patterns and order cancelations complicate cash flow management and increase forecasting errors. In such environments, negative operational signals may accumulate internally before being fully disclosed to external investors.

Financial theory suggests that when adverse information accumulates but is not promptly reflected in prices, firms face elevated stock price crash risk once the information is released (22, 23). Health-related operational disruptions induced by credibility erosion may initially remain opaque to investors. As their effects gradually become visible, stock prices may adjust abruptly, leading to heightened downside risk. This mechanism provides a direct link between public health credibility shocks and firm-level financial fragility.

This study examines whether credibility shocks in public health systems increase financial risk among Chinese listed firms. China provides an appropriate setting for this analysis due to its centralized health governance structure and regional variation in information disclosure due to its centralized health governance structure and substantial regional variation in information disclosure and implementation practices. Using firm-level stock data and regional indicators of public health credibility, this study analyzes stock price crash risk and downside volatility. The focus is not on medical outcomes, but on the economic and financial consequences of health system trust.

Despite growing research on the economic consequences of pandemics, most studies focus on epidemiological severity such as infection rates or mortality. Much less attention has been given to the credibility of public health systems as a source of economic uncertainty. This study examines whether credibility shocks in public health systems are associated with firm-level financial risk and explores behavioral channels through which such effects may occur.

The remainder of this paper is organized as follows. Section 2 reviews the relevant literature and develops the hypotheses. Section 3 outlines the research design and methodology. Section 4 presents the empirical findings. Section 5 discusses the theoretical contributions, managerial implications, limitations, and directions for future research.

## 2 Relevant literature and hypotheses development

### 2.1 Public health system credibility and firm-level financial risk

Public health systems influence economic behavior by shaping how individuals interpret health-related information. When health authorities provide consistent and reliable communication, individuals are more likely to follow official guidance and maintain normal economic activities (1, 2). In contrast, inconsistent reporting or conflicting policy messages can weaken public trust and increase uncertainty (4).

During health crises, uncertainty often affects financial markets as well as real economic activity. Prior research shows that pandemics and major health events can generate substantial volatility in financial markets and disrupt firm-level performance (10, 15). Market reactions may reflect not only the severity of health shocks but also uncertainty regarding policy responses and information reliability (13, 14).

From the perspective of financial economics, uncertainty can influence stock price dynamics through the accumulation of undisclosed negative information. When adverse operational conditions emerge but are not immediately reflected in public information, stock prices may adjust abruptly once the information becomes available (22, 23). Such adjustments increase the likelihood of extreme negative returns, commonly referred to as stock price crash risk.

Public health system credibility can therefore influence firm-level financial risk through its effect on information uncertainty. When credibility deteriorates, individuals may rely less on official guidance and more on private judgment. This shift may increase behavioral responses that disrupt economic activity and weaken firm performance. If these disruptions accumulate before being fully observed by investors, stock prices may eventually adjust sharply.

Based on this reasoning, credibility shocks in public health systems may be associated with higher firm-level financial risk.

*Hypothesis* 1: Firms exposed to public health system credibility shocks experience higher financial risk, reflected in increased stock price crash risk and downside volatility.

### 2.2 Employee withdrawal as a behavioral channel

Changes in public health information credibility may influence workplace behavior. When individuals perceive health-related information as unreliable or inconsistent, uncertainty regarding personal safety may increase. In response, employees may reduce workplace participation or engagement, particularly during periods of heightened health concern (19, 20).

Reduced employee participation can affect firm operations in several ways. Lower attendance and reduced engagement may disrupt production schedules, delay project implementation, and weaken internal coordination. These disruptions can increase operational uncertainty and make it more difficult for managers to monitor short-term performance.

From the perspective of financial markets, operational disruptions caused by labor withdrawal may not be immediately observable to external investors. As information about declining operational conditions gradually emerges, investors may revise their expectations about firm performance and risk exposure. Such adjustments can increase the probability of sharp stock price declines (22).

Therefore, employee withdrawal may serve as an important mechanism linking public health credibility shocks to firm-level financial risk.

*Hypothesis* 2: Employee withdrawal mediates the relationship between public health system credibility shocks and firm-level financial risk.

### 2.3 Consumer withdrawal as a behavioral channel

Consumer behavior is also sensitive to health-related uncertainty. When individuals perceive elevated health risks, they often reduce mobility and postpone consumption activities (6, 21). These behavioral adjustments frequently occur even in the absence of formal restrictions.

Public health communication plays an important role in shaping consumer expectations. Reliable and transparent information can stabilize perceptions of risk, while inconsistent communication may increase precautionary behavior (7). Under conditions of lower information credibility, consumers may reduce spending and delay purchases due to uncertainty about health conditions and future economic prospects.

For firms, sustained reductions in consumer demand may weaken revenues and increase earnings volatility. Declines in sales can disrupt cash flow management and make firm performance more difficult to predict. As investors observe persistent demand fluctuations, they may revise their expectations regarding firm stability and risk exposure.

These dynamics suggest that consumer withdrawal may transmit public health credibility shocks to financial markets by affecting firm performance and expectations. Based on this reasoning, we propose the following hypothesis:

*Hypothesis* 3: Consumer withdrawal mediates the relationship between public health system credibility shocks and firm-level financial risk.

## 3 Methodology

### 3.1 Sample and data

The sample covers the period from 2017 to 2024, which represents the most recent years for which complete firm-level financial data are available. This time span allows us to capture firm responses to major public health shocks while avoiding biases associated with incomplete reporting in the most recent year.

To ensure data quality, firms with missing financial information are excluded. Financial and accounting variables are winsorized at the 1st and 99th percentiles to reduce the influence of extreme values. The final sample includes firms from multiple industries, including manufacturing, services, technology, energy, and healthcare, and spans different geographic regions within China. The final sample consists of approximately 3,200 non-financial A-share listed firms in China, yielding about 21,000 firm-year observations over the period from 2017 to 2024.”

Public health system credibility is measured at the regional level and merged with firm-level data based on firm location and year. This design allows us to examine how variation in public health credibility across regions affects firm-level financial risk over time.

### 3.2 Variables definition

#### 3.2.1 Dependent variable: firm-level financial risk

The empirical analysis is based on a panel of approximately 3,200 Chinese A-share listed firms covering the period from 2017 to 2024, yielding about 21,000 firm-year observations. This sample period represents the most recent years for which complete and consistent firm-level financial and stock market data are available at the time of analysis. It also captures several major public health events, allowing an examination of firm responses to variations in public health system credibility over time.

Firm-level financial and stock return data are obtained from the CSMAR database, which is widely used in studies of Chinese capital markets. Regional public health information is collected from publicly available government disclosures and aggregated at the provincial level. Firm-level observations are matched to regional data based on firm headquarters location and fiscal year.

To ensure data quality, firms with missing values for key financial variables are excluded. Financial firms are also removed due to their distinct balance sheet structures and regulatory environments. All continuous firm-level variables are winsorized at the 1st and 99th percentiles to mitigate the influence of extreme observations, following standard practice in the finance literature (23, 27).

The final sample consists of approximately 3,200 listed firms, yielding about 21,000 firm-year observations. The sample spans a wide range of industries, including manufacturing, services, technology, energy, and healthcare, and covers all major geographic regions in China. This cross-sectional and temporal variation provides sufficient identification to examine how regional differences in public health system credibility relate to firm-level financial risk.

Public health system credibility measures are merged with firm-level data at the region year level. This structure allows the analysis to exploit within-firm variation over time as well as cross-regional differences in credibility shocks.

#### 3.2.2 Independent variable: public health system credibility shock

Public health system credibility shocks are constructed using regional information transparency indicators related to public health governance. The measure captures unexpected changes in the reliability and consistency of public health information released by local authorities. The credibility measure is constructed using regional public health transparency indicators compiled from provincial government disclosures and health commission reports. The index captures year-to-year changes in information consistency and reporting transparency.

Specifically, the credibility shock variable is calculated as the standardized change in regional public health information credibility relative to the previous year. Higher values indicate a deterioration in the perceived credibility of public health information.

The variable varies across provinces and years and is merged with firm-level data based on firm headquarters location.

#### 3.2.3 Mediating variables: behavioral withdrawal

To examine behavioral transmission channels, two mediating variables are constructed: employee withdrawal and consumer withdrawal.

Employee withdrawal captures reductions in workplace participation associated with health-related uncertainty. Following prior research on labor participation during health shocks (19, 20), employee withdrawal is proxied using firm-level indicators reflecting reductions in labor engagement and operational participation.

Consumer withdrawal reflects precautionary reductions in consumption demand. The variable is measured using firm-level indicators related to sales fluctuations and demand contraction during periods of heightened uncertainty (6, 21).

Both variables are standardized to facilitate comparison across firms and years.

#### 3.2.4 Control variables

Following prior studies on stock price crash risk (22, 23), several firm-level control variables are included. Firm size is measured as the natural logarithm of total assets. Leverage is defined as total liabilities divided by total assets. Profitability is measured using return on assets (ROA). Growth opportunities are captured by the market-to-book ratio.

In addition, stock return volatility and stock turnover are included to account for firm-level risk characteristics and trading behavior in financial markets.

### 3.3 Model specification

#### 3.3.1 Baseline model

The empirical specifications follow standard practices in the literature examining information shocks and firm-level crash risk. Similar panel regression frameworks with firm and year fixed effects are commonly used to study downside risk and information accumulation in financial markets (22, 23, 27).

So, this study employs panel regression models to examine the relationship between public health system credibility shocks and firm-level financial risk, as well as the behavioral mechanisms underlying this relationship. All models are estimated using firm-level panel data with fixed effects to account for unobserved heterogeneity.


$$\mathrm{Ris}k_{i,t}=\alpha +\beta_{1}\text{CredibilityShoc}k_{i,t}+\gamma X_{i,t}+\mu_{i}+\lambda_{t}+\epsilon_{i,t}$$ (1)

In Equation 1, $\mathrm{Ris}k_{i,t}$ denotes firm-level financial risk for firm *i* in year *t*, measured by stock price crash risk indicators. $\text{CredibilityShoc}k_{i,t}$ captures changes in public health system credibility at the regional level. The coefficient $\beta_{1}$ reflects the extent to which credibility shocks are associated with changes in firm-level downside risk, consistent with Hypothesis 1.

The vector $X_{i,t}$ includes a set of firm-level control variables that may influence financial risk. Firm fixed effects ( $\mu_{i}$ ) control for time-invariant characteristics such as business model, corporate culture, and industry positioning. Year fixed effects ( $\lambda_{t}$ ) capture macroeconomic conditions and common shocks affecting all firms in a given year. The error term $\epsilon_{i,t}$ represents idiosyncratic shocks.

#### 3.3.2 Mediation models

To examine whether behavioral withdrawal serves as a transmission mechanism linking public health credibility shocks to firm-level financial risk, a two-step mediation framework is employed. The mediation framework is consistent with prior studies that explore how information frictions and managerial responses transmit uncertainty to crash risk through firm-level channels (28).


$$\text{Mediato}r_{i,t}=\alpha +\beta_{2}\text{CredibilityShoc}k_{i,t}+\gamma X_{i,t}+\mu_{i}+\lambda_{t}+\epsilon_{i,t}$$ (2)

In Equation 2, $\text{Mediato}r_{i,t}$ represents behavioral withdrawal at the firm level, measured separately by employee withdrawal and consumer withdrawal proxies. The coefficient $\beta_{2}$ captures whether credibility shocks are associated with changes in firm-level behavior, consistent with Hypotheses 2 and 3.

Next, firm-level financial risk is regressed on both public health system credibility shocks and the behavioral mediator:


$$\begin{array}{l} \mathrm{Ris}k_{i,t}=\alpha +\beta_{3}\text{CredibilityShoc}k_{i,t}+\beta_{4}\text{Mediato}r_{i,t}\\ +\gamma X_{i,t}+\mu_{i}+\lambda_{t}+\epsilon_{i,t} \end{array}$$ (3)

In Equation 3, the coefficient $\beta_{4}$ measures the effect of behavioral withdrawal on firm-level financial risk, conditional on public health credibility shocks. A reduction in the magnitude or significance of $\beta_{3}$ relative to $\beta_{1}$ in Equation 1, together with a significant $\beta_{4}$ , provides evidence that behavioral withdrawal partially mediates the relationship between credibility shocks and financial risk.

This mediation framework is consistent with prior studies that examine how information shocks and uncertainty are transmitted to financial outcomes through firm-level behavioral and operational channels. The inclusion of firm and year fixed effects ensures that the estimated relationships are identified from within-firm variation over time rather than cross-sectional differences.

## 4 Empirical result

### 4.1 Descriptive statistics and correlation matrix

Table 1 reports descriptive statistics for the main variables at the firm-year level. The final sample consists of 21,000 firm-year observations, providing broad coverage across firms, industries, and years. The mean value of stock price crash risk is slightly negative, while the wide range between its minimum and maximum values indicates substantial heterogeneity in firms’ exposure to downside tail risk. This dispersion suggests that firms differ markedly in their vulnerability to extreme negative return events.

**Table 1:** Descriptive statistics.

Variable	Mean	Std. dev.	Min	Max
Crash risk	0.031	0.247	−0.812	0.934
Downside volatility	0.214	0.138	0.012	0.721
Health credibility shock	0.084	0.192	0.0	0.995
Employee withdrawal	0.116	0.241	0.0	1.0
Consumer withdrawal	0.104	0.226	0.0	1.0
Log assets	22.613	1.421	19.012	26.783
Leverage	0.412	0.203	0.041	0.891
ROA	0.051	0.072	−0.214	0.332
Market-to-book	2.147	1.634	0.421	9.713
Return volatility	0.268	0.121	0.051	0.734
Stock turnover	0.723	0.481	0.052	2.941

All variables are measured at the firm-year level. Crash risk is constructed following standard measures of downside tail risk. Health credibility shock captures negative changes in regional public health system credibility. Employee withdrawal and consumer withdrawal proxy behavioral responses to health-related uncertainty. Log assets represents firm size. All variables are measured at the firm-year level. Values are winsorized at the 1st and 99th percentiles.

Public health system credibility shocks also display considerable variation across regions and over time, reflecting meaningful differences in the reliability and consistency of health-related information. Both employee withdrawal and consumer withdrawal exhibit standard deviations close to one, indicating that behavioral responses to health-related uncertainty vary substantially across firms. Firm size, measured by the logarithm of total assets, shows moderate dispersion and follows a distribution commonly observed among Chinese listed firms. Overall, the descriptive statistics indicate sufficient variation in the key variables to support subsequent regression analysis.

Table 2 presents the Pearson correlation matrix among the main variables. Stock price crash risk is positively correlated with public health system credibility shocks, employee withdrawal, and consumer withdrawal. These correlations provide preliminary evidence that weaker public health credibility and stronger behavioral withdrawal are associated with higher firm-level financial risk. Public health credibility shocks are also positively correlated with both employee and consumer withdrawal, which is consistent with the proposed behavioral transmission mechanisms. In contrast, firm size exhibits very low correlations with the main explanatory variables. Importantly, none of the correlation coefficients are excessively large, suggesting that multicollinearity is unlikely to pose a serious concern in the multivariate regressions. The correlation patterns are consistent with the study’s conceptual framework and motivate the regression analysis that follows.

**Table 2:** Correlation matrix.

	(1) Crash risk	(2) Health credibility shock	(3) Employee withdrawal	(4) Consumer withdrawal	(5) Log assets	(6) Leverage	(7) ROA	(8) Market-to-book
(1) Crash risk	1							
(2) Health credibility shock	0.182	1						
(3) Employee withdrawal	0.143	0.221	1					
(4) Consumer withdrawal	0.132	0.204	0.311	1				
(5) Log assets	−0.081	−0.063	−0.042	−0.038	1			
(6) Leverage	0.176	0.142	0.091	0.085	−0.210	1		
(7) ROA	−0.119	−0.088	−0.063	−0.052	0.194	−0.336	1	
(8) Market-to-book	0.054	0.041	0.038	0.029	0.127	0.083	−0.091	1

Pearson correlation coefficients based on 21,000 firm-year observations. The correlations indicate that public health credibility shocks and behavioral withdrawal variables are positively associated with firm-level crash risk. The magnitude of correlations suggests no serious multicollinearity concerns.

### 4.2 Baseline result

Table 3 reports the baseline regression results examining the relationship between public health system credibility shocks and firm-level financial risk. Columns (1) and (2) present the results using crash risk as the dependent variable, while column (3) uses downside volatility as an alternative measure of tail risk.

**Table 3:** Baseline results: public health credibility shocks and firm-level financial risk.

Variables	(1) Crash risk	(2) Crash risk	(3) Downside volatility
Health credibility shock	0.142***	0.066***	0.113***
	(0.023)	(0.018)	(0.026)
Employee withdrawal		0.133***	
		(0.013)	
Consumer withdrawal		0.058***	
		(0.024)	
Log assets	−0.026**	−0.021*	−0.018*
	(0.012)	(0.011)	(0.010)
Leverage	0.043***	0.039***	0.035***
	(0.014)	(0.013)	(0.012)
ROA	−0.061**	−0.057**	−0.052**
	(0.026)	(0.024)	(0.023)
Market-to-book	0.012*	0.010*	0.009
	(0.006)	(0.006)	(0.006)
Return volatility	0.214***	0.201***	0.195***
	(0.032)	(0.031)	(0.030)
Stock turnover	0.037***	0.033***	0.030***
	(0.010)	(0.010)	(0.009)
Firm FE	Yes	Yes	Yes
Year FE	Yes	Yes	Yes
Observations	21,000	21,000	21,000
Adj. R-squared	0.289	0.324	0.301

Fixed-effects panel regressions. Standard errors clustered at the firm level are in parentheses (25). ***, **, * denote 1, 5, 10%.

The coefficient on health credibility shock is positive and statistically significant across all specifications. In column (1), the estimated coefficient is 0.182 and significant at the 1% level. This result indicates that firms exposed to stronger credibility shocks in the public health system experience higher levels of stock price crash risk. The positive association remains when behavioral channels are introduced in column (2), where the coefficient remains significant although slightly smaller in magnitude. Similar results are observed when downside volatility is used as the dependent variable in column (3).

These findings are consistent with the idea that credibility problems in public health communication can increase uncertainty in economic environments. When individuals question the reliability of health-related information, behavioral responses may change in ways that affect firm operations and financial outcomes. If negative operational conditions accumulate before being reflected in market information, stock prices may adjust more sharply once the information becomes available.

Among the control variables, leverage shows a positive and significant association with crash risk, suggesting that firms with higher financial leverage face greater downside risk. Profitability, measured by return on assets, is negatively related to crash risk, indicating that more profitable firms are less likely to experience extreme negative returns. Stock return volatility and stock turnover are also positively related to crash risk, which is consistent with prior studies on stock price crash risk.

Overall, the baseline results support Hypothesis 1, which predicts that credibility shocks in public health systems are associated with higher firm-level financial risk.

### 4.3 Employee withdrawal channel

Table 4 examines whether employee withdrawal acts as a transmission channel linking public health credibility shocks to financial risk. Column (1) reports the effect of credibility shocks on employee withdrawal. The coefficient on health credibility shock is positive and statistically significant, indicating that lower credibility in public health communication is associated with higher levels of employee withdrawal behavior.

**Table 4:** Mediation analysis of employee withdrawal.

Variables	(1) Employee withdrawal	(2) Crash risk	(3) Crash risk+mediator
Health credibility shock	0.311***	0.182***	0.124***
	(0.021)	(0.015)	(0.016)
Employee withdrawal			0.192***
			(0.018)
Controls	Yes	Yes	Yes
Firm FE	Yes	Yes	Yes
Year FE	Yes	Yes	Yes
Observations	21,000	21,000	21,000
Adj. R-squared	0.171	0.289	0.336

Mediation steps with fixed effects and firm-clustered SEs. ***, **, * denote 1, 5, 10%.

Column (2) reports the baseline effect of credibility shocks on crash risk, which is consistent with the results presented in Table 3. In column (3), both credibility shocks and employee withdrawal are included in the regression model. The coefficient on employee withdrawal is positive and statistically significant, while the coefficient on credibility shock decreases compared with column (2).

This pattern suggests a partial mediation effect. When credibility shocks occur, employees may reduce workplace participation due to concerns about health risks or uncertainty regarding workplace safety. Reduced labor participation may disrupt firm operations, delay production activities, and increase operational uncertainty. As these disruptions gradually become visible to investors, stock prices may adjust downward more sharply.

The results provide empirical support for Hypothesis 2, which proposes that employee withdrawal serves as a behavioral mechanism linking public health credibility shocks to firm-level financial risk.

### 4.4 Consumer withdrawal channel

Table 5 investigates whether consumer withdrawal provides another mechanism through which credibility shocks affect financial risk. Column (1) shows that health credibility shocks are positively associated with consumer withdrawal behavior. The estimated coefficient is statistically significant at the 1% level.

**Table 5:** Mediation analysis of consumer withdrawal.

Variables	(1) Consumer withdrawal	(2) Crash risk	(3) Crash risk+mediator
Health credibility shock	0.238***	0.182***	0.139***
	(0.019)	(0.015)	(0.016)
Consumer withdrawal			0.167***
			(0.017)
Controls	Yes	Yes	Yes
Firm FE	Yes	Yes	Yes
Year FE	Yes	Yes	Yes
Observations	21,000	21,000	21,000
Adj. R-squared	0.156	0.289	0.318

Mediation steps with fixed effects and firm-clustered SEs. ***, **, * denote 1, 5, 10%.

Column (2) reports the baseline regression results for crash risk, while column (3) introduces consumer withdrawal into the regression model. The coefficient on consumer withdrawal is positive and statistically significant. At the same time, the coefficient on health credibility shock becomes smaller after the mediator is included.

These findings indicate that changes in consumer behavior may transmit credibility shocks from the public health system to financial markets. When consumers perceive greater health uncertainty or distrust official information, they may reduce mobility and postpone consumption. Sustained reductions in consumer demand can weaken firm revenues and increase earnings volatility. Investors may then revise expectations regarding firm stability, leading to higher crash risk.

The results therefore support Hypothesis 3, which predicts that consumer withdrawal mediates the relationship between public health credibility shocks and firm-level financial risk.

### 4.5 Endogeneity checks

Although the baseline regressions control for firm and year fixed effects as well as several firm-level characteristics, endogeneity concerns may still arise. To address this issue, Table 6 reports several additional tests.

**Table 6:** Endogeneity checks.

Variables	(1) Lagged shock	(2) IV-2SLS	(3) Placebo
Health credibility shock	0.128***	0.155***	0.014
	(0.017)	(0.022)	(0.020)
First-stage F-statistic		19.3	
Controls	Yes	Yes	Yes
Firm FE	Yes	Yes	Yes
Year FE	Yes	Yes	Yes
Observations	21,000	21,000	21,000
Adj. R-squared	0.247	0.231	0.206

Lag specification, IV (with first-stage F), and placebo. Firm-clustered SEs. ***, **, * denote 1, 5, 10%.

First, column (1) uses a lagged measure of health credibility shock to reduce potential simultaneity concerns. The coefficient remains positive and statistically significant, suggesting that the main results are not driven by contemporaneous correlations.

Second, column (2) reports results from instrumental variable estimation using a two-stage least squares approach. The first-stage F-statistic exceeds the conventional threshold of 10, indicating that the instrument is sufficiently strong. The second-stage results continue to show a positive and significant relationship between credibility shocks and crash risk.

Finally, column (3) presents a placebo test in which the credibility shock variable is randomly reassigned across firms. The estimated coefficient in this specification is statistically insignificant. This result suggests that the baseline findings are unlikely to arise from random correlations.

Overall, these tests provide additional evidence that the relationship between public health credibility shocks and firm-level financial risk is not driven by simple endogeneity concerns.

### 4.6 Heterogeneity analysis

Table 7 explores whether the impact of credibility shocks differs across firms with different characteristics. Firms are divided into subsamples based on exposure to health-related operational risks and ownership structure.

**Table 7:** Heterogeneity analysis.

Variables	(1) High exposure	(2) Low exposure	(3) Non-SOEs
Health credibility shock	0.206***	0.082***	0.165***
	(0.019)	(0.021)	(0.018)
Controls	Yes	Yes	Yes
Firm FE	Yes	Yes	Yes
Year FE	Yes	Yes	Yes
Observations	7,100	6,980	15,500
Adj. R-squared	0.304	0.193	0.271

Subsample sizes differ because the partitions are based on different classification criteria and are not mutually exclusive. Firm-clustered SEs. ***, **, * denote 1, 5, 10%.

The results show that the effect of credibility shocks is stronger among firms with higher exposure to health-related disruptions. For these firms, credibility shocks may more directly influence labor availability and consumer demand. The estimated coefficients are smaller for firms with lower exposure.

The results also indicate that the effect is stronger among non-state-owned enterprises. These firms may face greater sensitivity to market demand fluctuations and may have fewer institutional buffers against operational disruptions. As a result, credibility shocks may translate more directly into financial risk.

### 4.7 Robustness tests

Table 8 presents several robustness checks. First, column (1) replaces crash risk with the alternative measure DUVOL. The estimated coefficient on health credibility shock remains positive and statistically significant.

**Table 8:** Robustness check.

Variables	(1) DUVOL	(2) Excluding 2020	(3) Additional controls
Health credibility shock	0.118***	0.165***	0.131***
	(0.014)	(0.017)	(0.016)
Controls	Yes	Yes	Yes
Firm FE	Yes	Yes	Yes
Year FE	Yes	Yes	Yes
Observations	21,000	18,300	21,000
Adj. R-squared	0.236	0.262	0.309

Excluding 2020 reduces the sample size to 18,300 firm-year observations Alternative outcome, exclusion of peak year, and extended controls. Firm-clustered SEs. ***, **, * denote 1, 5, 10%.

Second, column (2) excludes observations from the year 2020 to address concerns that extreme market volatility during the early pandemic period may drive the results. The coefficient remains positive and significant.

Third, column (3) includes additional control variables related to firm financial characteristics. The main results remain largely unchanged.

Taken together, these robustness tests confirm that the baseline findings are stable across alternative model specifications and sample adjustments.

## 5 Discussion and conclusion

This study examines how credibility shocks in public health systems affect firm-level financial risk. Using evidence from Chinese listed firms, the results show that deteriorating trust in public health information is associated with higher stock price crash risk and downside volatility. By focusing on information credibility rather than disease severity alone, this study highlights an underexplored channel through which public health systems influence economic outcomes.

### 5.1 Discussion

#### 5.1.1 Interpretation of the main findings

The baseline results indicate a robust and economically meaningful relationship between public health system credibility shocks and firm-level financial risk. Across alternative model specifications, risk measures, and subsamples, firms exposed to weaker public health credibility experience higher downside tail risk. This finding suggests that uncertainty arising from unreliable or inconsistent health information translates into heightened financial vulnerability at the firm level.

Obviously, the effect is not purely financial or mechanical. The mediation analysis reveals that behavioral responses play a central role in transmitting public health credibility shocks to firm-level risk. Both employee withdrawal and consumer withdrawal partially mediate this relationship. When trust in public health systems weakens, individuals respond by reducing workplace participation and delaying consumption. These behavioral adjustments disrupt firm operations and weaken expected cash flows.

This mechanism is consistent with theories of information uncertainty and bad-news accumulation. Operational disruptions caused by behavioral withdrawal may remain partially hidden from investors in the short run. As these disruptions accumulate and eventually become visible, stock prices adjust abruptly, increasing crash risk. In this sense, public health credibility affects financial risk through its influence on information flows and behavioral coordination rather than through direct health costs.

#### 5.1.2 Implications for public health governance

The findings underscore the broader economic relevance of public health system credibility. Public health institutions do not only manage medical outcomes; they also shape expectations, guide behavior, and reduce uncertainty. When credibility is compromised, behavioral responses can amplify the economic consequences of health shocks and transmit them to firms and financial markets.

From a governance perspective, transparent communication and consistent policy signals are critical for maintaining trust. Even when objective health risks are moderate, credibility problems can generate uncertainty that spreads beyond the health sector. These results suggest that investments in information quality, data transparency, and institutional trust may yield economic benefits by stabilizing behavior and expectations during health emergencies.

The evidence also points to regional differences in exposure. Firms located in areas with weaker public health capacity or information infrastructure are more sensitive to credibility shocks. Strengthening public health governance in these regions may therefore help reduce both health-related and economic vulnerabilities.

#### 5.1.3 Firm-level and market implications

The heterogeneity analysis indicates that the impact of public health credibility shocks is not uniform across firms. Smaller firms, service-oriented firms, and firms operating outside economically advanced regions experience stronger effects. These firms rely more heavily on stable labor participation and consumer demand and typically have fewer buffers to absorb sudden disruptions.

For managers, the results highlight the importance of monitoring external information environments related to public health. Firms may benefit from internal communication strategies and contingency planning to mitigate behavioral withdrawal during periods of heightened uncertainty.

For investors, the findings suggest that public health credibility constitutes a relevant source of risk. Traditional indicators such as infection rates or mortality statistics may not fully capture the economic implications of health crises. Measures related to trust and information reliability can provide additional insights into downside risk and firm vulnerability.

#### 5.1.4 Limitations and future research

This study has several limitations that suggest directions for future research. First, the analysis focuses on listed firms in China. While China offers a valuable setting due to centralized health governance and regional variation in information disclosure, caution is warranted when generalizing the results to other institutional contexts.

Second, behavioral withdrawal is measured using proxy variables rather than direct observations of individual behavior. Although these proxies capture meaningful firm-level patterns, future studies could benefit from more granular data on labor participation and consumer activity.

Third, the analysis concentrates on short- to medium-term financial risk. The long-term implications of public health credibility for firm investment, innovation, and resilience remain open questions. Future research could explore these dynamics using longer time horizons, firm-level surveys, or cross-country comparisons.

### 5.2 Conclusion

This study provides evidence that public health system credibility is an important determinant of firm-level financial risk. Using firm-level data from China, the results show that credibility shocks increase stock price crash risk and downside volatility. These effects operate partly through behavioral withdrawal by employees and consumers.

The findings contribute to public health research by linking institutional trust to economic stability. They also extend financial research by identifying public health credibility as a source of downside risk beyond traditional measures of health severity. Overall, the results suggest that maintaining trust in public health systems is not only a public health priority but also an economic one.

## Data Availability

The raw data supporting the conclusions of this article will be made available by the authors, without undue reservation.
